# Supplementary material for: HIVprotI: an integrated web based platform for prediction and design of HIV proteins inhibitors
Source: J Cheminform. 2018 Mar 9;10:12. doi: 10.1186/s13321-018-0266-y (PMC5845081; doi:10.1186/s13321-018-0266-y)
Supplement: Supplementary file 2 — Additional file 2. Source code of HIVProtI web server. [file 13321_2018_266_MOESM2_ESM.zip › HIVprotI_Source-code/hivprotI/jsmol/jsv_jme.htm]

JSpecView/JSME/nmrdb/NIH-Resolver Demo II


|  |  |
| --- | --- |
| Enable Editing | Name It! Capture Image |

Enter an identifier such as a chemical name or SMILES string, or enable editing and draw a compound.
Then click 
to see a **simulated** spectrum. Or load your own spectrum by dragging a file into the blue box or using
a right-click to access the pop-up menu.

info
clear
spectrum
integration
(drag to zoom; right-click for menu; note that OH and NH may not be shown)

RSC SpectraSchool Assigned Spectra Examples:
IPA-1HNMR
IPA-13CNMR
TCB-1HNMR
TCB-13CNMR
carvone-IR
carvone-MS

This page illustrates how we can use JSME (the JavaScript Molecular Editor)
  
along with JSpecView to quickly get a simulated spectrum for a compound of our choice.
  
Note that the three applets can be combined easily in any combination.
  
  
JSmol on this page calls servers in Frederick, Maryland (NIH resolver, for name-to-structure)
  
and Lausanne, Switzerland (nmrdb, for structure-to-spectrum)
  
  
Draw a chemical structure
  
or search for a chemical identifier such as **caffeine** or *CCOCC*,
  
then press .
  
  
Note that these spectra are *just predictions.*
  
They may differ significantly from actual NMR spectra.

credits:
JSmol and page development: Bob Hanson
JSME: Peter Ertl, Bruno Bienfait
JSpecView: Robert Lancashire, Bob Hanson
nmrdb: Luc Patiny
NIH Resolver: Markus Sitzmann
Java2Script: Zhou Renjian
